# MiRNA‐145‐5p expression and prospective molecular mechanisms in the metastasis of prostate cancer

**DOI:** 10.1049/syb2.12011

**Published:** 2021-02-01

**Authors:** Zhi‐Guang Huang, Yu Sun, Gang Chen, Yi‐Wu Dang, Hui‐Ping Lu, Juan He, Ji‐Wen Cheng, Mao‐Lin He, Sheng‐Hua Li

**Affiliations:** ^1^ Department of Pathology The First Affiliated Hospital of Guangxi Medical University Nanning Guangxi Zhuang Autonomous Region P.R. China; ^2^ Division of Spinal Surgery The First Affiliated Hospital of Guangxi Medical University Nanning Guangxi Zhuang Autonomous Region P.R. China; ^3^ Department of Urology The First Affiliated Hospital of Guangxi Medical University Nanning Guangxi Zhuang Autonomous Region P.R. China

## Abstract

The clinicopathological implication and prospective molecular mechanisms of miRNA‐145‐5p in the metastasis of prostate cancer (PCa) stand unclear. Herein, it is found that miRNA‐145‐5p expression was remarkably reduced in 131 cases of metastatic PCa than 1371 cases of localised ones, as the standardised mean differences (SMD) was −1.26 and the area under the curve (AUC) was 0.86, based on miRNA‐chip and miRNA‐sequencing datasets. The potential targets of miRNA‐145‐5p in metastatic PCa (*n* = 414) was achieved from the intersection of miRNA‐145‐5p transfected metastatic PCa cell line data, differential expression of metastatic PCa upregulated genes and online prediction databases. TOP2A was screened as one of the target hub genes by PPI network analysis, which was adversely related to miRNA‐145‐5p expression in both metastatic PCa (*r* = −0.504) and primary PCa (*r* = −0.281). Gene‐chip and RNA‐sequencing datasets, as well as IHC performed on clinical PCa samples, showed consistent upregulated expression of TOP2A mRNA and protein in PCa compared with non‐PCa. The expression of TOP2A mRNA was also significantly higher in metastatic than localised PCa with the SMD being 1.72 and the AUC of sROC being 0.91. In summary, miRNA‐145‐5p may participate in PCa metastasis by binding TOP2A and be useful as a biomarker for the detection of metastatic PCa.

## 1 INTRODUCTION

Prostate cancer (PCa) is one of the most common malignant tumours in humans. Among all male cancers, PCa is also the second most frequent cause of cancer‐related death, accounting for 20% of newly diagnosed cancers and an estimated 29,430 deaths according to 2020 cancer statistics [1]. Although urologists have made great progress in the diagnosis and therapy of PCa, the metastasis of this cancer remain serious problems that adversely affect the quality of life and survival time of patients with PCa [2, 3, 4, 5, 6, 7]. Therefore, clarifying the mechanisms of PCa metastasis is essential, as knowledge of these mechanisms may offer new insights into metastatic PCa diagnosis and therapy.

MicroRNAs (miRNAs) are small (about 22 nucleotides long), endogenous, non‐coding single‐stranded eukaryotic RNAs that can influence the expression of their target genes post‐transcriptionally [8, 9, 10, 11, 12, 13, 14]. Abnormal expression of miRNA‐145‐5p has a reported involvement in multiple cancers, including hepatocellular carcinoma, oesophageal carcinoma and lung cancer. MiRNA‐145‐5p plays crucial parts in the tumorigenesis and deterioration of hepatocellular carcinoma by targeting RAB18 [15]. Downregulation of miRNA‐145‐5p expression increased the risk of oesophageal carcinoma, and it was closely associated with the cell differentiation, lymph node invasion and distant metastasis [16]. In lung cancer, miRNA‐145‐5p modulated the epithelial‐mesenchymal transition (EMT) and promoted metastatic infiltration by aiming ZEB2, which controls lung cancer cell migration [17]. MiRNA‐145‐5p plays a crucial role in the onset, development and treatment of PCa [18, 19, 20, 21, 22, 23, 24]. However, researches on the role of miRNA‐145‐5p in the metastasis of PCa have been limited. Through the analysis of 14 miRNAs in 14 localised PCa and 5 metastatic PCa, Leite et al. found that miRNA‐145‐5p was downregulated in metastatic PCa [25]. In another study by Leite et al., the results also demonstrated that miRNA‐145‐5p expression was reduced in 6 metastatic PCa than 18 localised PCa [26]. Peng et al. performed miRNA chip analysis on 6 localised PCa and 7 metastatic PCa, showing that miRNA‐145‐5p expression was reduced in metastatic PCa, and further confirmed the result in 16 localised PCa and 13 metastatic PCa using real‐time PCR method; overexpression of miRNA‐145‐5p could reduce the invasion and migration ability of PC‐3 cells [27]. However, these results were investigated through a small sample sizes (*n* < 20), without verification with a larger sample sizes, which might reduce the credibility of the results. Moreover, the clinicopathological implication and prospective molecular mechanisms of miRNA‐145‐5p in metastatic PCa have not been elucidated.

A comprehensive valuation of the expression and distinguishing potential of miRNA‐145‐5p in metastatic PCa is given, conducted by estimating standard mean deviations (SMDs) and summary receiver operating characteristics (SROCs) based on miRNA‐chip and miRNA‐sequencing datasets. Gene ontology (GO) and Kyoto Encyclopedia of Genes and Genomes (KEGG) pathways were also generated to investigate the latent molecular characterisations involving miRNA‐145‐5p target genes in metastatic PCa. Potential hub target genes were screened with protein–protein interaction (PPI) network analyses, and TOP2A having the highest degree of connectivity among the hub genes was selected for in‐depth research via the Spearman's correlation analysis, estimating SMDs and SROCs and immunohistochemistry (IHC) of clinical PCa samples.

## 2 MATERIALS AND METHODS

### 2.1 Data collection of miRNA‐145‐5p expression in metastatic and primary PCa

We collected the miRNA‐145‐5p expression data for metastatic and primary PCa from miRNA‐chip and miRNA‐sequencing datasets. The search terms used for The Cancer Genome Atlas (TCGA), ArrayExpress, Sequence Read Achieve (SRA), Gene Expression Omnibus (GEO), Oncomine databases as well as the existing literature, were provided by our previous study concerning another miRNA in PCa [28]. Studies were counted in if: (1) the studies incorporated two groups (metastatic PCa group vs. localised PCa group or primary PCa group vs. non‐PCa group) and (2) the studies were based on human tissues, cell lines or body fluids. The studies were excluded if: (1) no miRNA‐145‐5p expression data were obtained and (2) the studies had fewer than three samples. The expression data for miRNA‐145‐5p were normalised using log2 conversion.

### 2.2 Putative target genes of miRNA‐145‐5p in metastatic PCa

Putative target genes of miRNA‐145‐5p were derived from the following three evaluations.

#### 2.2.1 MiRNA‐145‐5p transfected metastatic PCa cell line data

MiRNA‐chip and miRNA‐sequencing datasets were selected as qualified studies if they showed gene expression profiles post miRNA‐145‐5p transfection or silencing in metastatic PCa cell line. Genes with the standard log2FC > 1 or log2FC  <  −1 were filtered as genes directly influenced by the change of miRNA‐145‐5p.

#### 2.2.2 Differential expression of metastatic PCa upregulated genes

The search formula for the gene‐chip and RNA‐sequencing datasets was as mentioned above [28]. The inclusion conditions were the following: (1) The studies involved gene expression data for both a metastatic PCa and a localised PCa group and (2) the study samples were from human tissues, body fluids or cell lines. The exclusion criteria were: (1) Only lncRNA, circular RNA or miRNA expression data were obtained and (2) the studies had fewer than three samples. A total of 10 studies were finally included. The limma R package was leveraged to examine the differentially expressed genes (DEGs) in the gene‐chips. The DESeq2 R language package was used to screen the DEGs in the RNA sequencing. The DEGs threshold was set as an adjusted *p*‐value < 70.05 and |log2FC| > 1. Herein, the expression of miRNA‐145‐5p was downregulated in metastatic PCa, so the upregulated genes in metastatic PCa were more likely to be the targets of miRNA‐145‐5p. Upregulated genes were screened from the DEGs, and the genes that occurred at least three times were selected for subsequent analysis.

#### 2.2.3 Online prediction databases

The miRNAWalk2.0 program [29] was applied to anticipate the supposed target genes of miRNA‐145‐5p. Genes identified by at least five prediction tools among 12 were screened for further study. The intersections of these three sources were considered to represent the targets of miRNA‐145‐5p.

### 2.3 GO, KEGG and PPI analyses of potential miRNA‐145‐5p targets in metastatic PCa

The functions and pathways involving the miRNA‐145‐5p target genes were identified using GO and KEGG analysis as well as the database for Annotation, Visualization and Integrated Discovery (DAVID) 6.8. The GO terms and KEGG signal pathways with *p*‐value<0.05 were presented. Search Tool for the Retrieval of Interacting Genes (STRING) and Cytoscape software 3.6.1 were applied to construct a PPI network for miRNA‐145‐5p target genes. Hub genes were selected based on the degree of connectivity among miRNA‐145‐5p targets. Among the hub genes, TOP2A having the highest degree of connectivity was selected for further study.

### 2.4 Relationship between miRNA‐145‐5p and TOP2A

The miRNA‐145‐5p and TOP2A expression data in metastatic and primary PCa were downloaded from the TCGA database. The expression data were normalised by log2(*x* + 1). Spearman's correlations were computed between miRNA‐145‐5p and TOP2A in both metastatic and primary PCa. Furthermore, the relationships between miRNA‐145‐5p and TOP2A expression levels in different cancers were evaluated using StarBase v3.0. Negative relationships with *p* < 0.05 were statistically significant.

### 2.5 The clinical significance of TOP2A in the metastasis of PCa

#### 2.5.1 Data collection of TOP2A expression in metastatic and primary PCa

Relevant gene‐chip and RNA‐sequencing datasets providing TOP2A expression data were collected using the afore‐mentioned search terms for PCa. The inclusion criteria were: (1) the studies enrolled two groups (metastatic PCa group vs. localised PCa group or primary PCa group vs. non‐PCa group) and (2) the studies were based on human tissues, body fluids or cell lines. The exclusion criteria were the following: (1) no TOP2A expression data were present and (2) the study had fewer than three samples. TOP2A expression data were normalised by log2‐transformation.

#### 2.5.2 Determination of protein expression of TOP2A in PCa samples by IHC

Four tissue microarrays (PRD1021, PRC1021, PRC481 and PRC961) were purchased from Fanpu (Guilin, China) including 160 PCa tissues from patients aged between 47 and 91 years, as well as 61 non‐PCa tissues aged between 54 and 85 years. IHC was conducted as the manufacturers' protocols guided. Rabbit monoclonal anti‐TOP2A (1:100 diluted; catalog no. ab109524, Abcam, Cambridge, MA, USA) was the first antibody for IHC. The intensity and the percentage of positive cells were used to calculate an immunoreactive score (IRS) for each sample by multiplying the two scores as previously reported [30].

### 2.6 Statistical analysis

The standard mean deviation (SMD) with 95% confidential interval (CI) was used to estimate the differential expression of miRNA‐145‐5p and TOP2A between two independent groups using stata14.0 software (Stata Corporation, College Station, TX, USA). The pooled effect was determined by a random‐effect model if heterogeneity occurred between the studies (*p* < 0.05 or *I* ^2^ > 50%); otherwise, a fixed‐effects model was applied (*p* > 0.05 or *I* ^2^ < 50%). The cause of heterogeneity was explored by subgroup analysis and sensitivity analysis. Begg's test and egger's test were used to detect any publication bias in the included studies. The sensitivity and specificity of each study were determined by creating a receiver operating characteristic (ROC) curve and calculating the cut‐off values. A summary receiver operating characteristic (SROC) curve was produced to assess the distinguishing potential of miRNA‐145‐5p and TOP2A between two independent groups using stata14.0. Scatter diagrams were generated with GraphPad Prism5.0 (GraphPad Software, Inc., La Jolla, CA, USA) to show the expression trends for miRNA‐145‐5p and TOP2A between two independent groups. The statistics were analysed using SPSS22.0 (SPSS, IBM, Chicago, IL, USA), and the results were shown as mean ± standard deviation (SD). The differences between two independent groups were compared using independent *t*‐tests. Survival analysis was performed by the Kaplan–Meier method and log‐rank test. *p* < 0.05 was the cut‐off to be statistically significant. A flow chart of design is presented in Figure S1.

## 3 RESULTS

### 3.1 The clinical significance of miRNA‐145‐5p in the metastasis of PCa

**FIGURE 1:**
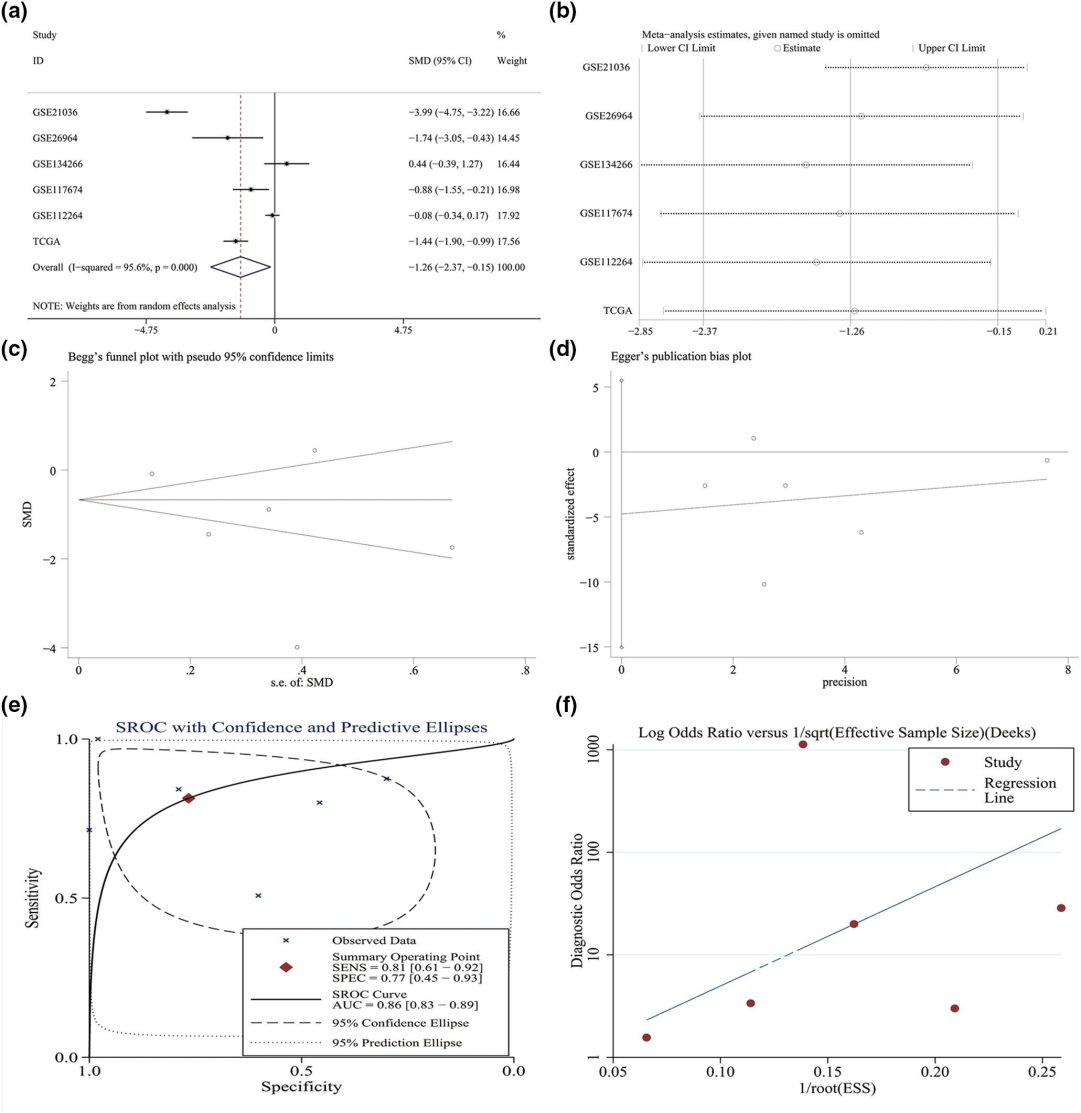
The expression level and discrimination potential of miRNA‐145‐5p in metastatic PCa. (a) Forest plot showing the combined SMD of −1.26 (−2.37 to −0.15), indicating that the expression of miRNA‐145‐5p in metastatic PCa is lower compared with that of localised PCa. (b) Sensitivity analysis showing the combined SMD is stable. (c) Begg's test showing no publication bias (p > 0.05). (d) Egger's test showing no publication bias (p > 0.05). (e) SROC curve assessing the discrimination potential of miRNA‐145‐5p in metastatic PCa. (f) Funnel chart showed no publication bias (p > 0.05)

**FIGURE 2:**
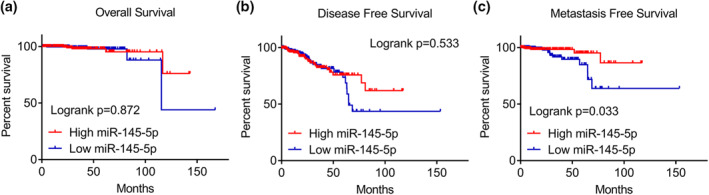
Kaplan–Meier analysis of miRNA‐145‐5p of PCa. (a) no statistical relationship was found between miRNA‐145‐5p and OS rate (*p* = 0.872). (b) no statistical relationship was found between miRNA‐145‐5p and DFS rate (*p* = 0.533). (c) PCa with downregulated miRNA‐145‐5p had a shorter MFS time than did the PCa with upregulated miRNA‐145‐5p (*p* = 0.033)

A total of six studies that contained miRNA‐145‐5p expression data were considered eligible (Table 1). The data extracted from the six studies showed a lower miRNA‐145‐5p expression in 131 metastatic PCa samples than in 1371 localised PCa samples (SMD = −1.26, 95% CI: −2.37 to −0.15, *I* ^2^ = 95.6%, *p* = 0.000) (Figure 1a). Because the samples are all from the tissues, we only conducted a sensitivity analysis to seek the source of heterogeneity, which indicated that no study had a significant influence on the whole study cohort (Figure 1b). No publication bias was observed (Figure 1c,d). The potential of miRNA‐145‐5p for distinguishing metastatic PCa from localised PCa was assessed by SROC analysis, which showed area under the curve (AUC) value of 0.86 (0.83–0.89) (Figure 1e). No publication bias was observed (Figure 1f). The scatter diagrams and ROC curves of miRNA‐145‐5p between metastatic PCa and localised PCa of each study were visualised in Figures S2 and S3. These data indicated a dramatic downregulation of miRNA‐145‐5p and a moderate potential of miRNA‐145‐5p for distinguishing metastatic PCa from localised PCa. We also verified the miRNA‐145‐5p expression between PCa and non‐PCa. A total of 26 studies were obtained (Table S1). As expected, we found that miRNA‐145‐5p expression was remarkably reduced in 1429 PCa samples as compared with 544 non‐PCa samples, as the standardised mean differences (SMD) was −0.30 and AUC was 0.76 (Figure S4). The association between miRNA‐145‐5p and clinical characteristics was analysed in PCa patients using the TCGA database. Downregulated miRNA‐145‐5p was significantly associated with pathological T, N and M stages, Gleason score and recurrence (Table S2). Survival analysis demonstrated the PCa with downregulated miRNA‐145‐5p had shorter metastasis‐free survival (MFS) time than did the PCa with upregulated miRNA‐145‐5p, while no statistical relationship was found between miRNA‐145‐5p and overall survival (OS) rate or disease‐free survival (DFS) rate (Figure 2).

**TABLE 1:** The means and standard deviations of miR‐145‐5p expression values in LPCa and MPCa based on six studies

Study	Country	Year	Sample	MPCa			LPCa		
			type	*N*	*M*	SD	*N*	*M*	SD
GSE21036	USA	2010	Tissue	14	10.947	1.324	99	14.163	0.711
GSE26964	China	2011	Tissue	7	7.552	3.577	6	12.385	1.263
GSE134266	China	2019	Tissue	8	2.460	0.510	20	2.331	0.144
GSE117674	Canada	2018	Tissue	19	17.847	0.489	19	18.303	0.543
GSE112264	Japan	2018	Tissue	63	2.884	2.401	746	3.095	2.494
TCGA	NA	NA	Tissue	20	11.721	0.827	481	12.914	0.827

Abbreviations: LPCa, localised PCa; *M*, mean; miR, microRNA; MPCa; metastatic PCa; *N*, number; PCa, prostate cancer; SD, standard deviation; TCGA, The Cancer Genome Atlas.

### 3.2 Putative target genes of miRNA‐145‐5p in metastatic PCa

#### 3.2.1 miRNA‐145‐5p transfected metastatic PCa cell line data

Examination of the GSM610397 (the bone metastatic PCa cell line PC‐3) and GSM610398 (the lymph node metastasis PCa cell line LNCap) data with the standard log2FC  <  −1, which included gene expression variations after the transfection of miRNA‐145‐5p mimic in metastatic PCa cell line, gave a total of 8365 and 7603 genes, respectively. After removing the duplicates, 12,031 genes were determined as candidates influenced by miRNA‐145‐5p.

#### 3.2.2 Differential expression of metastatic PCa upregulated genes

Screening of a total of 10 studies identified the differential expression of upregulated genes. In total, 1460 standard genes that occurred at least three times among the differentially expressed upregulated genes were selected for subsequent analysis.

#### 3.2.3 Online prediction databases

**FIGURE 3:**
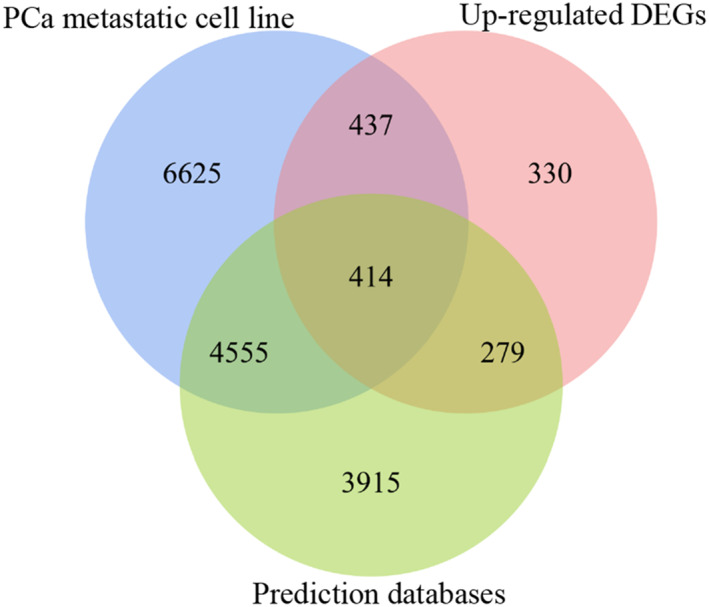
A total of 414 genes were selected as potential target genes of miRNA‐145‐5p

In total, 9163 predicted target genes emerged using at least five prediction tools. These were gathered and the overlap of the three sources was used to obtain an accurate evaluation of the targets of miRNA‐145‐5p. Ultimately, 414 genes obtained from the overlapping results from the three sources, were gathered for subsequent analysis (Figure 3).

### 3.3 GO, KEGG and PPI analyses of the putative targets of miRNA‐145‐5p in metastatic PCa

**FIGURE 4:**
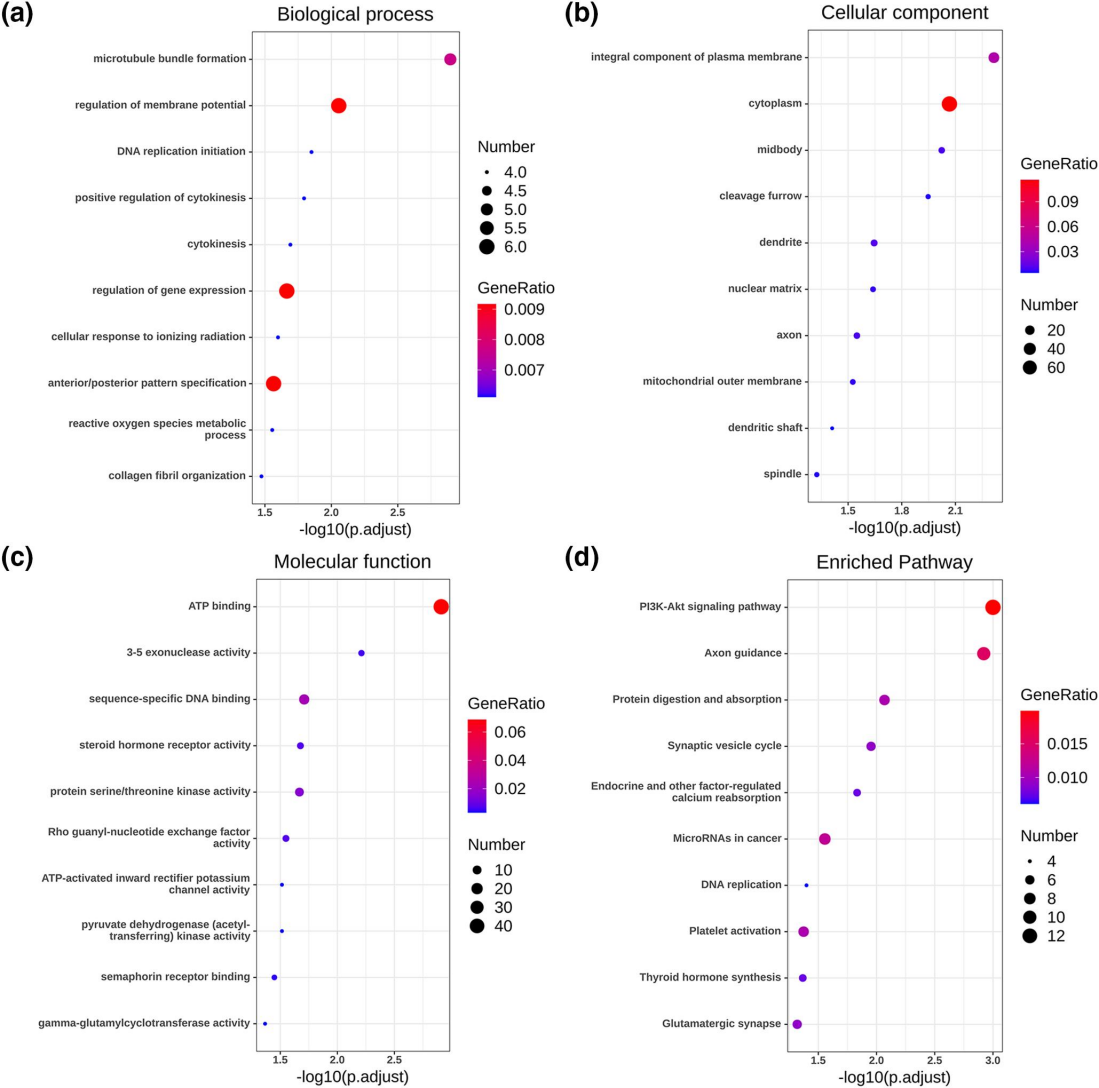
Functional and pathway enrichment analyses of the target genes of miRNA‐145‐5p. (a) Biological processes. (b) Cellular component. (c) Molecular function. (d) Kyoto Encyclopedia of Genes and Genomes analysis

**FIGURE 5:**
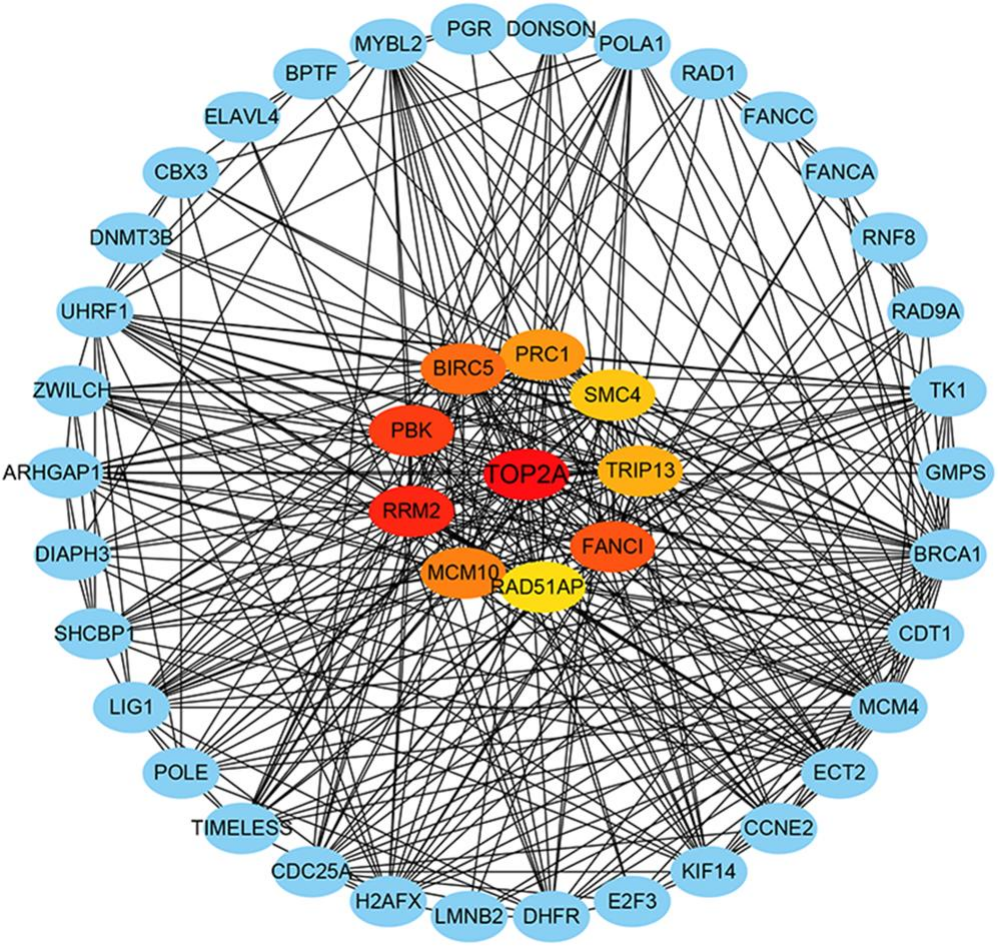
PPI network analysis screening results for the hub target genes of miRNA‐145‐5p in metastatic PCa

GO enrichment using DAVID showed that the 414 intersected potential targets were enriched most significantly in the subsequent pathways: microtubule bundle formation, regulation of membrane potential and DNA replication initiation in the biological process (BP) (Figure 4a); integral component of plasma membrane, cytoplasm and midbody in the cellular component (CC) (Figure 4b); ATP binding, 3′−5′ exonuclease activity and sequence‐specific DNA binding in the molecular function (MF) (Figure 4c). The KEGG pathway analysis suggested that the PI3K‐Akt signalling pathway was most significant (Figure 4d). The PPI network analysis defined ten genes (TOP2A, RRM2, PBK, BIRC5, PRC1, FANC1, MCM10, TRIP13, SMC4 and RAD51AP) as the hub genes in metastatic PCa, and TOP2A having the highest degree of connectivity was selected for further study (Figure 5).

### 3.4 Relationship between miRNA‐145‐5p and TOP2A

**FIGURE 6:**
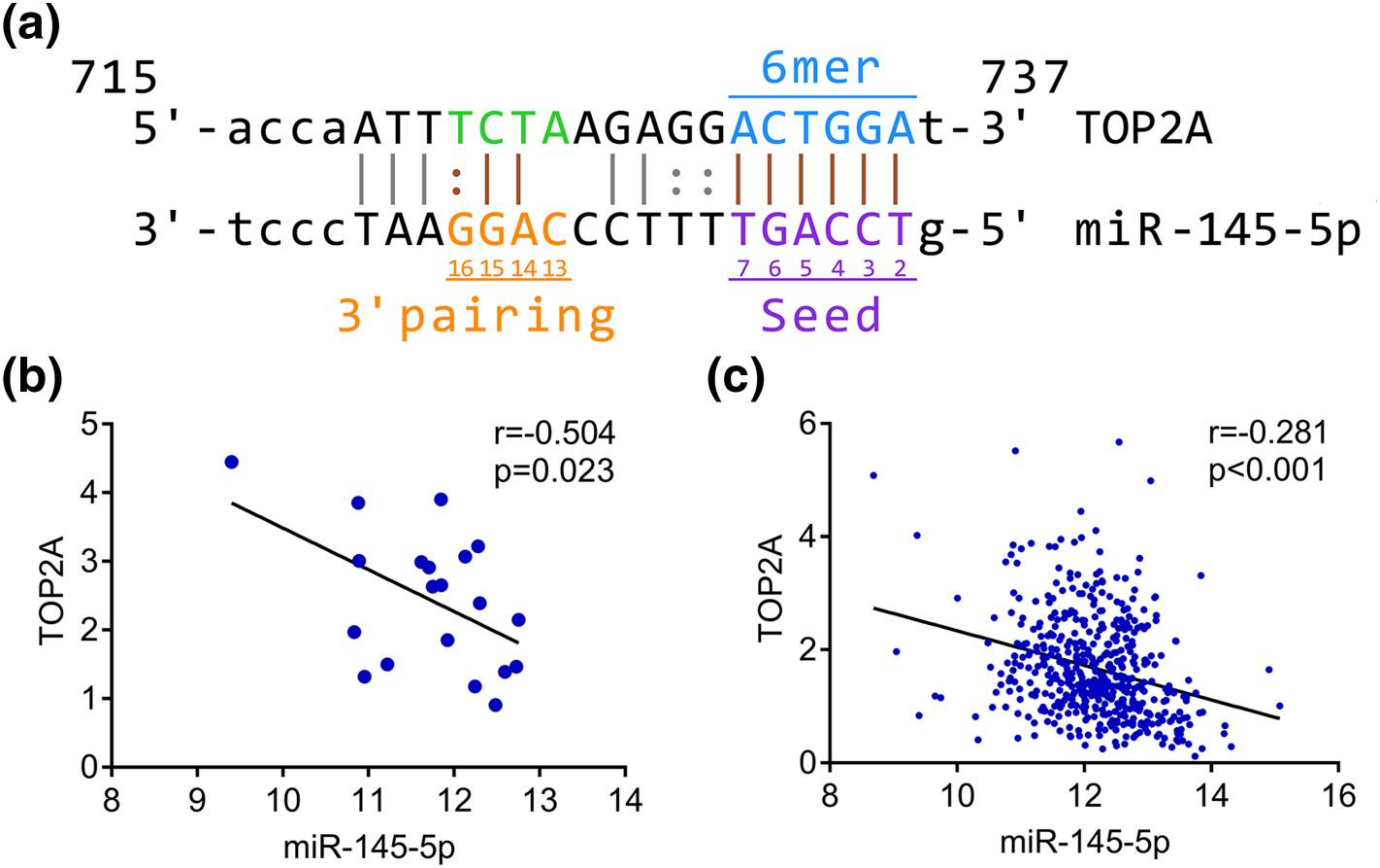
Relationship between miRNA‐145‐5p and TOP2A. (a) Complementary base sequences of miRNA‐145‐5p and TOP2A. (b) Correlation analysis between TOP2A and miRNA‐145‐5p in metastatic PCa. (c) Correlation analysis between TOP2A and miRNA‐145‐5p in primary PCa

We observed the base‐complementary pairing between miRNA‐145‐5p and TOP2A using miRwalk 2.0 (Figure 6a). Spearman's correlation analysis was applied to detect the relationships between miRNA‐145‐5p and TOP2A in both metastatic and primary PCa. The results showed that there were significantly reverse relationships between miRNA‐145‐5p and TOP2A in both metastatic PCa (*r* = −0.504, *P* = 0.023) and primary PCa (*r* = −0.281, *P* < 0.001) (Figure 6b,c). Furthermore, reverse relationships between miRNA‐145‐5p and TOP2A were observed in 14 different cancers (Figure S7).

### 3.5 The clinical significance of TOP2A in the metastasis of PCa

**FIGURE 7:**
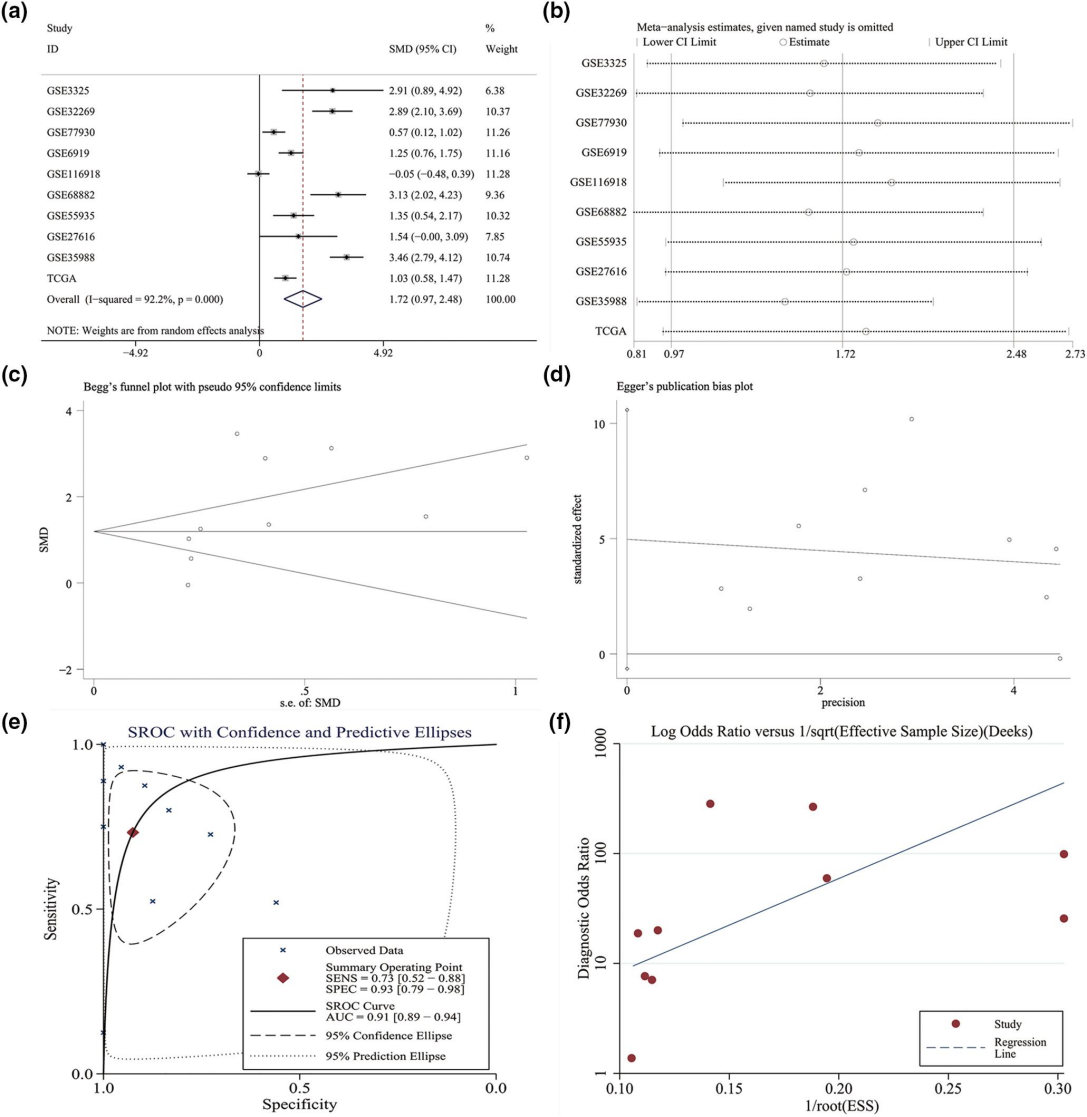
Expression level and discrimination potential of TOP2A in metastatic PCa. (a) Forest plot showing a combined SMD of 1.72 (0.97–2.48), indicating that the expression of TOP2A in metastatic PCa was higher compared with that of localised PCa. (b) Sensitivity analysis showing the combined SMD is stable. (c) Begg's test showing no publication bias (*p* > 0.05). (d) Egger's test showed no publication bias (*p* > 0.05). (e) SROC curve assessing the discrimination potential of CELSR3 in metastatic PCa. (f) Funnel chart showing no publication bias (*p* > 0.05)

**FIGURE 8:**
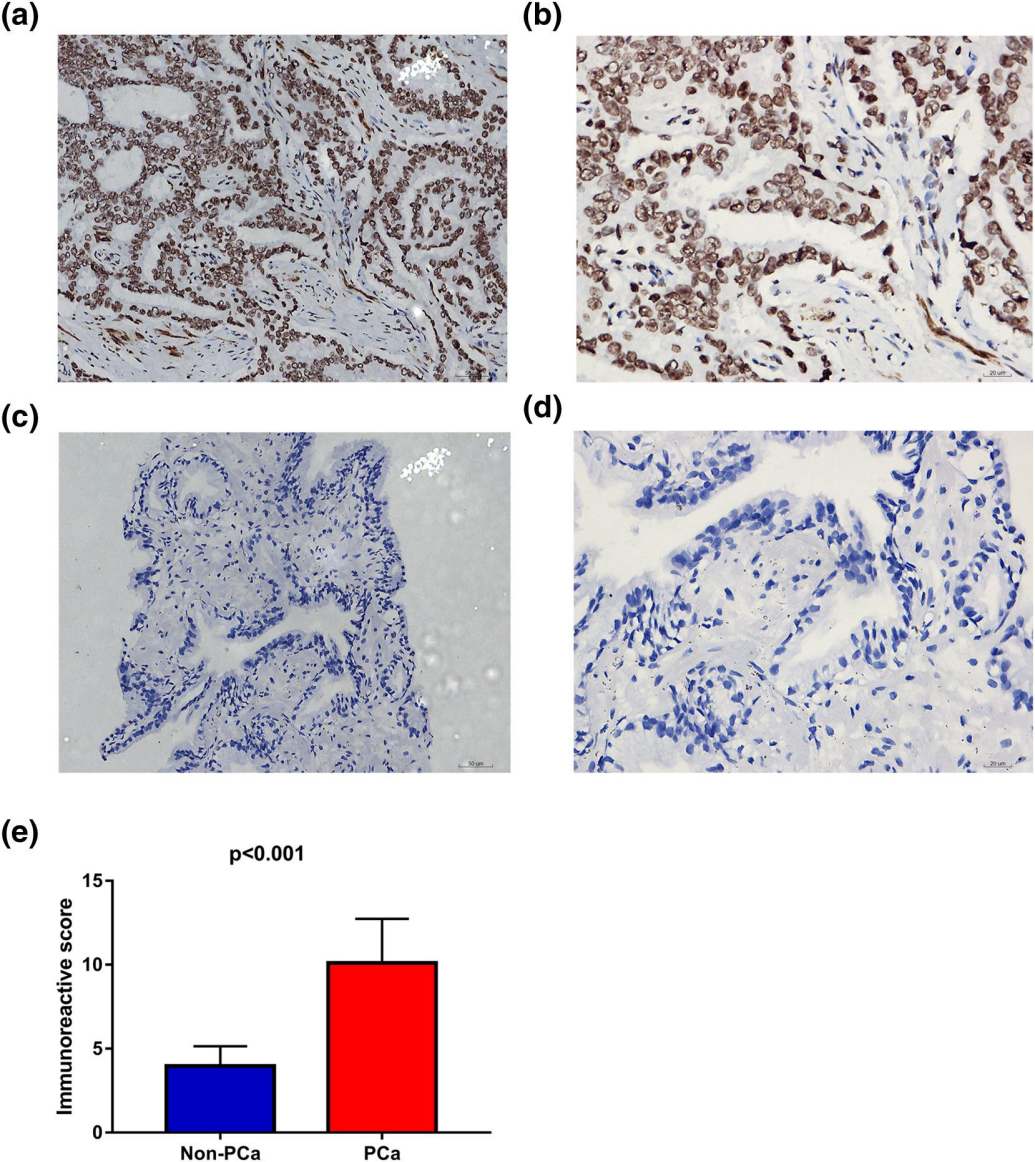
TOP2A protein expression in PCa and non‐PCa tissues by immunohistochemical staining. (a), (b) PCa tissues stained intensely for TOP2A (magnification, ×200 lower and ×400 upper). (c), (d) non‐ PCa tissues stain medium for TOP2A (magnification, ×200 lower and ×400 upper). (e) Histogram showing the expression of TOP2A proteins in PCa and non‐PCa tissues based on immunoreactive score

**FIGURE 9:**
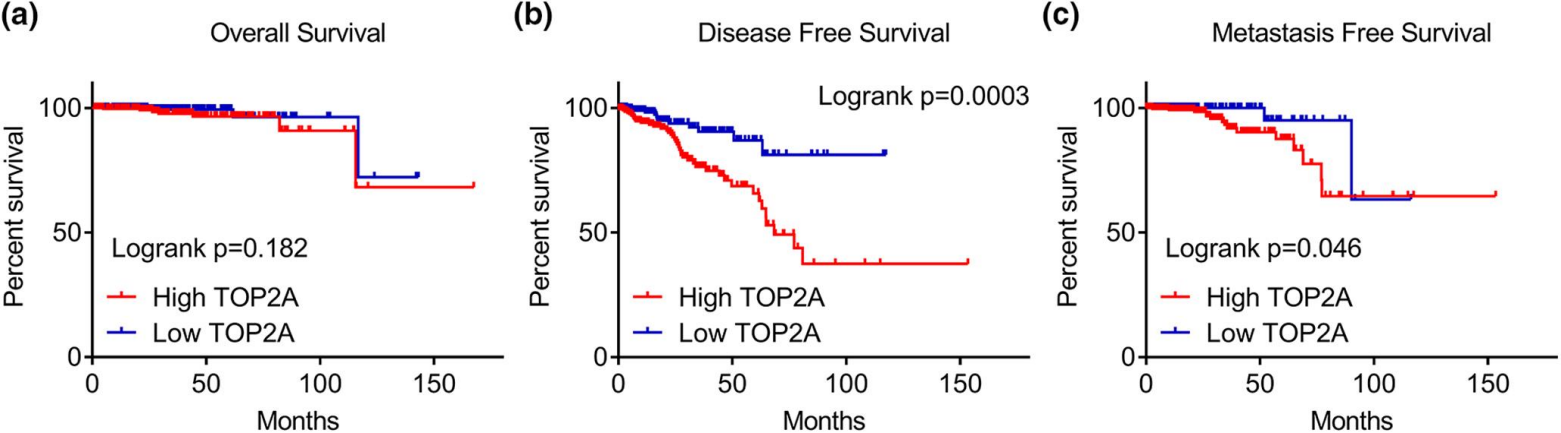
Kaplan–Meier analysis of TOP2A of PCa. (a) OS rate of PCa patients with high TOP2A versus low TOP2A expression was no difference (*p* = 0.182). (b) Statistical correlation was observed between TOP2A and DFS rate (*p* = 0.0003). (c) Statistical correlation was observed between TOP2A and MFS rate (*p* = 0.046)

A total of 10 studies that contained TOP2A expression data were considered eligible for the present study (Table 2). We applied a random‐effect model (*I* ^2^ = 81.0%, *P* = 0.001) to evaluate the combined SMD, which was 1.72 (0.97–2.48). This SMD value suggested that a higher TOP2A expression in 303 metastatic PCa samples than 943 localised PCa samples (Figure 7a). Because the samples are all from the tissues, we only conducted a sensitivity analysis, which indicated that no study had a significant influence on the whole study cohorts (Figure 7b). No publication bias was observed (Figure 7c,d). The potential of TOP2A to distinguish metastatic PCa from localised PCa was assessed by drawing SROC. The pooled AUC was 0.91 (0.89–0.94) (Figure 7e), and no publication bias was observed (Figure 7f). The scatter diagrams and ROC curves of TOP2A of each study were shown in Figures S5 and S6. Based on the above results, TOP2A was dramatically upregulated and had an outstanding distinguishing potential in metastatic PCa. We also verified the TOP2A expression between PCa and non‐PCa. The inclusion and exclusion criteria retrieved 19 studies (Table S3). TOP2A expression was significantly higher in 1231 PCa samples as compared to 537 non‐PCa samples with SMD being 0.68 and AUC being 0.82 (Figure S7). IHC staining demonstrated a remarkably increased TOP2A expression level in PCa tissues than in non‐PCa tissues (7.478 ± 3.386 vs. 5.959 ± 3.304, *P* < 0.001) (Figure 8). These results showed clear upregulation of TOP2A in PCa and a moderate potential of TOP2A expression for distinguishing between PCa and non‐PCa. The association between TOP2A and clinical characteristics was analysed in PCa patients using the TCGA database. TOP2A was significantly associated with age, pathological T, N and M stages, Gleason score and recurrence (Table S4). A statistical correlation was observed between TOP2A and DFS rate and MFS rate, while OS rate of PCa patients with high TOP2A versus low TOP2A expression was no difference (Figure 9).

**TABLE 2:** The means and standard deviations of TOP2A expression values in LPCa and MPCa based on 10 studies

Study	Country	Year	Sample	MPCa			LPCa		
			type	*N*	*M*	SD	*N*	*M*	SD
GSE3325	USA	2005	Tissue	4	10.267	1.908	5	6.234	0.800
GSE32269	USA	2011	Tissue	29	6.750	0.759	22	4.434	0.853
GSE77930	USA	2016	Tissue	149	10.083	1.267	22	9.372	1.161
GSE6919	USA	2007	Tissue	25	6.434	0.706	66	5.289	0.980
GSE116918	UK	2018	Tissue	22	2.859	0.364	225	2.874	0.328
GSE68882	USA	2015	Tissue	9	8.781	0.830	23	6.724	0.583
GSE55935	Norway	2014	Tissue	8	8.091	0.378	38	7.493	0.453
GSE27616	USA	2011	Tissue	4	4.767	1.378	5	3.203	0.614
GSE35988	USA	2012	Tissue	32	−0.292	0.329	59	−1.021	0.101
TCGA	NA	NA	Tissue	21	2.553	1.102	478	1.640	0.881

Abbreviations: LPCa, localised PCa; *M*, mean; MPCa; metastatic PCa; *N*, number; PCa, prostate cancer; SD, standard deviation; TCGA, The Cancer Genome Atlas.

## 4 DISCUSSION

A worldwide and extensive investigation of the expression level and distinguishing potential of miRNA‐145‐5p in metastatic PCa is provided. We observed that miRNA‐145‐5p was reduced in metastatic PCa, which had a moderate distinguishing potential in metastatic PCa. Putative miRNA‐145‐5p targets were derived from miRNA‐145‐5p transfected metastatic PCa cell line data, differential expression of metastatic PCa upregulated genes and online prediction databases, and 414 putative targets of miRNA‐145‐5p were screened. GO, KEGG and PPI network analyses were applied to identify a promising molecular mechanism involving miRNA‐145‐5p target genes. TOP2A had the highest connectivity in the PPI network analyses and was verified via the Spearman's correlation analysis, estimating SMDs and SROCs and IHC of clinical PCa samples.

Previous literature have reported that miRNA‐145‐5p as a tumour suppressor was low expression in PCa tissues, which is consistent with our result by calculating the pooled SMD in 1532 PCa tissue samples [31, 32, 33, 34, 35, 36, 37, 38, 39]. Xu et al. detected that miRNA‐145‐5p was increased in 60 urine samples of PCa patients than 37 urine samples of non‐PCa patients [40]. Paunescu et al. performed miRNA profiling in plasma of PCa patients and found miRNA‐145‐5p was upregulated in 14. plasma of PCa patients versus 15 controls [41]. Inconsistent with the results of previous studies, we found no statistical difference in 225 body fluids of PC samples versus 204 controls. Some studies reported that miRNA‐145‐5p was reduced in PCa cell lines [39,42,43], while our study showed that the expression of miRNA‐145‐5p did not show any statistical difference between the PC cell lines and non‐PCa cell lines. Therefore, the expression of miRNA‐145‐5p in PCa body fluids and cell lines needs further verification. Richardsen et al. found that miRNA‐145‐5p was not correlated to Gleason grade [44]. Our study indicated miRNA‐145‐5p was decreased in high Gleason grade patients (≥8) than low Gleason grade patients (≤7). For the relationship between miRNA‐145‐5p and prognosis, Larne et al. found the PCa with downregulated miRNA‐145‐5p had a shorter median survival time than did the PCa with upregulated miRNA‐145‐5p [19]. Avgeris et al. reported the loss of miRNA‐145‐5p expression resulted a shorter DFS time of PCa patients [31]. No statistical significance was found between miRNA‐145‐5p and OS rate or DFS rate in PCa patients using the TCGA database. Additional research is needed to further confirm the clinical effects of miRNA‐145‐5p in PCa.

Ren and colleagues found that low level of WT‐p53 accelerated the metastasis of PCa through inhibiting miRNA‐145‐5p expression to promote EMT [45]. In Guo et al.’s study, miRNA‐145‐5p was reported to target HEF1 repressing invasion and EMT in PCa [46]. MiRNA‐145‐5p and ZEB2 could form a negative feedback loop, which accelerated the progression and metastasis of PCa [47]. However, research on miRNA‐145‐5p expression in metastatic PCa is limited. Only a few studies with small sample size (*n* < 20) results indicated that miRNA‐145‐5p was reduced in metastatic PCa [25, 26, 27], and these results have not been verified by large sample. Herein, we observed downregulated miRNA‐145‐5p expression in 131 metastatic PCa samples than in 1371 localised PCa samples. Besides, miRNA‐145‐5p had good distinguishing potential in metastatic PCa, and downregulated miRNA‐145‐5p predicted poor MFS in PCa patients. MiRNA‐145‐5p might serve as a clinical biomarker in metastatic PCa.

We screened 414 miRNA‐145‐5p target genes using three different methods, which increased the reliability of the targets. The KEGG pathway analysis revealed the most significant pathway closely associated with metastatic PCa as PI3K‐Akt signalling pathway. Previous literature reported PI3K‐AKT signalling pathway activation was related to PCa metastasis [48, 49, 50, 51]. Two studies have shown that miRNAs (miRNA‐133a‐3p and miRNA‐188‐5p) suppressed PCa metastasis via inhibiting PI3K/AKT signalling pathway [52,53]. Although there is no research report on the connection between miRNA‐145‐5p and PI3K‐AKT signalling pathway in metastatic PCa, we believe that the role of miRNA‐145‐5p in PI3K/AKT signalling pathway is worth studying.

The results from the PPI network analysis identified 10 hub genes, and TOP2A having the highest degree of connectivity was selected as the potential targets for miRNA‐145‐5p. TOP2A had a significantly reverse relationship with miRNA‐145‐5p in both PCa and metastatic PCa. TOP2A encodes topoisomerase IIα, a nuclear enzyme that manipulates the DNA topological structure and cell cycle. This enzyme is a biomarker of cell proliferation in both tumour and non‐tumour cells. Recent studies have revealed that TOP2A plays a vital role in the genesis of many malignant tumours. TOP2A expression was upregulated in colon cancer than non‐cancer, and knockdown of TOP2A could inhibit cancer cell growth and invasion [54]. In breast cancer, TOP2A amplification was correlated with sensitivity to anthracycline‐based neoadjuvant chemotherapy, and TOP2A was identified as a predictive marker [55]. Pei et al. found a significant correlation between upregulation of TOP2A and pancreatic metastasis and with poorer survival in patients with pancreatic cancer [56]. Consistent with the results by calculating the pooled SMD in 1768 PCa samples and IHC of 221 clinical PCa samples, TOP2A has been reported to be upregulated in PCa, which accelerated the onset and progression of PCa via different biological mechanisms [57, 58, 59, 60, 61]. At present, the clinical significance of TOP2A is controversial. Malhotra et al. reported that TOP2A was not associated with pathologic T stage and Gleason grade [59]. In our study, TOP2A expression was increased in T3/4 and higher Gleason grade (≥8) patients. For the correlation between TOP2A and prognosis, several studies indicated that upregulated TOPA2 in patients was associated with a shorter time to biochemical recurrence [59, 61, 62]. Li et al. found that TOP2A was increased in secondary PCa and TOP2A overexpression resulted a shorter OS time of PCa patients. Amanda et al. reported that TOP2A overexpression was significantly related to OS rate of PCa patients [63]. The result showed that the OS rate of PCa patients with high TOP2A versus low TOP2A expression was no difference. The clinical significance of TOP2A in PCa needs further study.

For metastatic PCa, high expression of TOPA2 in PCa patients was associated with poorly differentiated primary disease, indicating that patients with high TOPA2 expression might be more prone to suffer a progressive and metastatic disease [62]. Xuefeng et al. reported that TOP2A was high expression in metastatic PCa compared with primary PCa, and TOP2A mediated EMT and cancer stem cells regulating metastasis of PCa [64]. An involvement of TOP2A in epigenetic regulation has been reported through EZH2, and TOP2A was increased in four murine metastatic PCa samples compared with three murine primary samples [65]. However, these studies were conducted through a small sample, which reduced the credibility of results. Herein, TOP2A was dramatically elevated in 303 metastatic PCa samples compared with 943 localised PCa samples. In addition, TOP2A had an outstanding distinguishing potential in metastatic PCa, and high expression of TOP2A predicted unfavourable MFS time in PCa patients. TOP2A may be useful as a biomarker for metastatic PCa.

Although we have obtained valuable findings, our research does have some limitations. First, the clinical significance of miRNA‐145‐5p in metastatic PCa have not been verified by our the clinical specimens from our institute. Second, we paid more attention to investigating the clinical significance and prospective molecular mechanisms of miRNA‐145‐5p in metastatic PCa; however, the biological functions of miRNA‐145‐5p and TOP2A in metastatic PCa and the relationship between miRNA‐145‐5p and TOP2A need to be further verified through both in vitro and in vivo experiments.

In conclusion, the results based on miRNA‐chip and miRNA‐sequencing datasets indicated that downregulation of miRNA‐145‐5p might promote PCa metastasis. TOP2A was determined as a potential target gene of miRNA‐145‐5p in metastatic PCa; this finding should be confirmed by further studies. In addition, miRNA‐145‐5p and TOP2A might serve as clinical biomarkers in metastatic PCa.

## Supporting information

Supplementary material 1

Supplementary material 2

Supplementary material 3

Supplementary material 4

Supplementary material 5

Supplementary material 6

Supplementary material 7

Supplementary material 8

Supplementary material 9

Supplementary material 10

Supplementary material 11

Supplementary material 12
