# Supplementary figures and images for: MiRNA‐145‐5p expression and prospective molecular mechanisms in the metastasis of prostate cancer
Source: IET Syst Biol. 2021 Feb 1;15(1):1–13. doi: 10.1049/syb2.12011 (PMC8675798; doi:10.1049/syb2.12011)

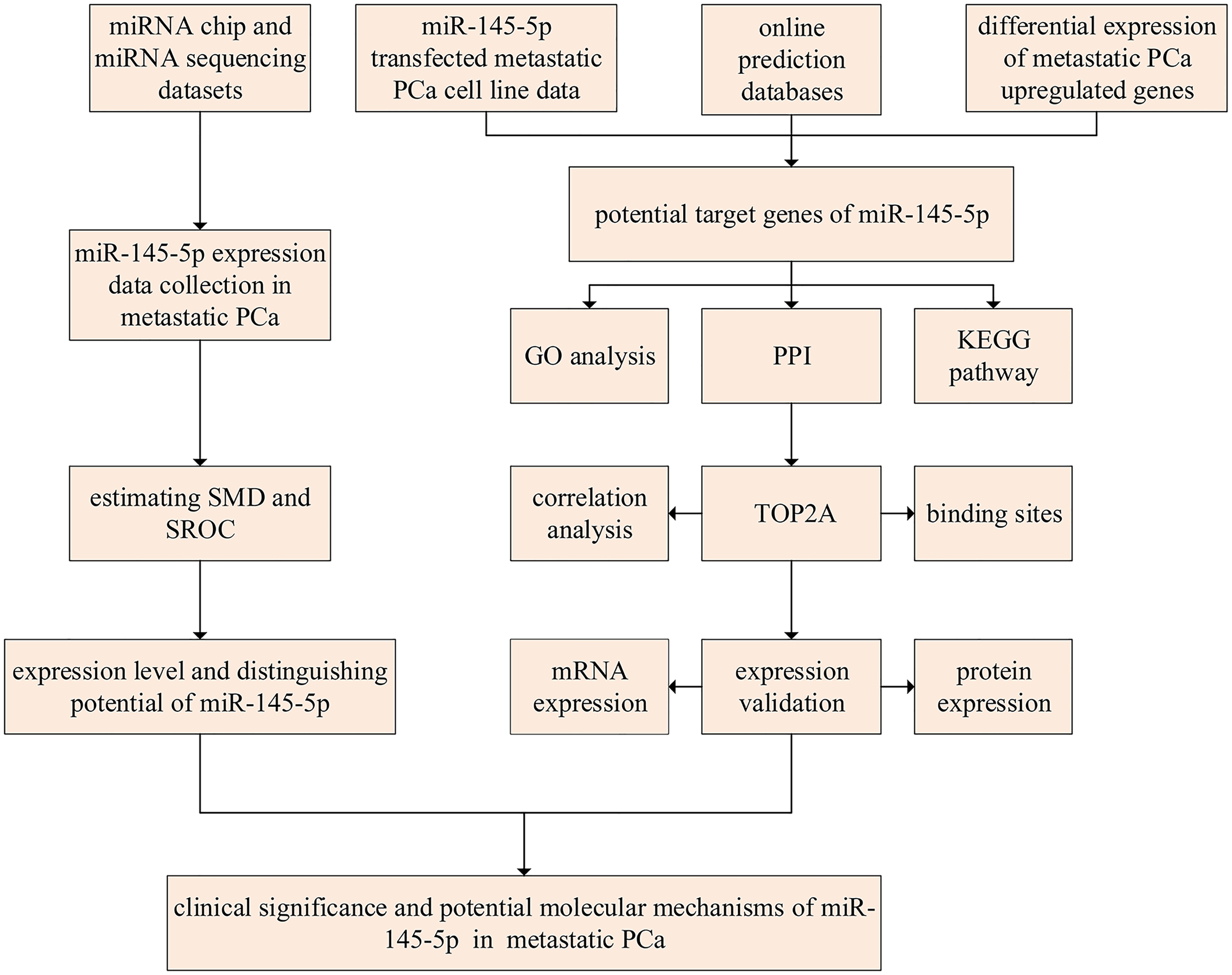

Supplement: Supplementary file 1 — Supplementary material 1 [file SYB2-15-1-s001.tif]

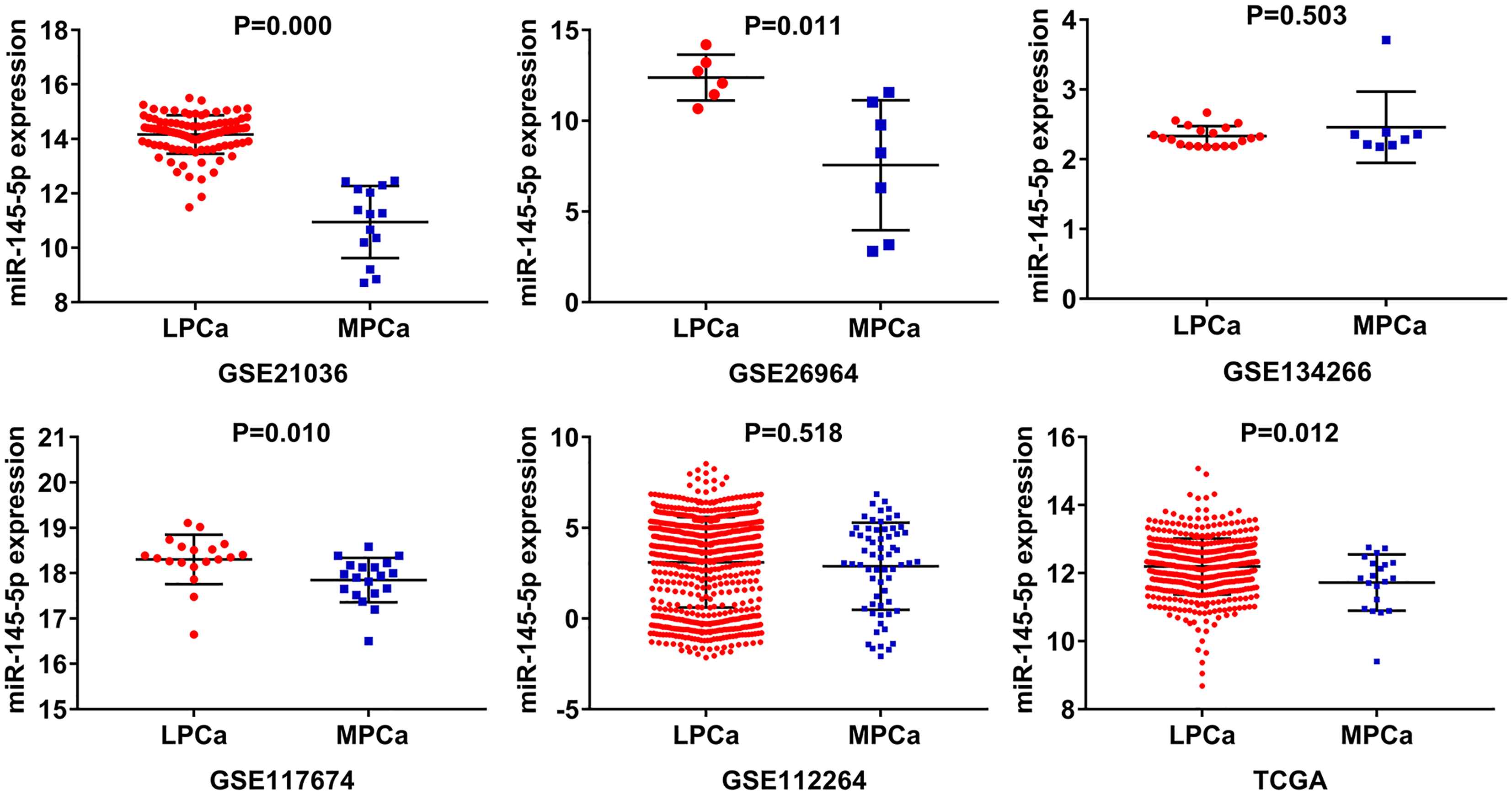

Supplement: Supplementary file 2 — Supplementary material 2 [file SYB2-15-1-s002.tif]

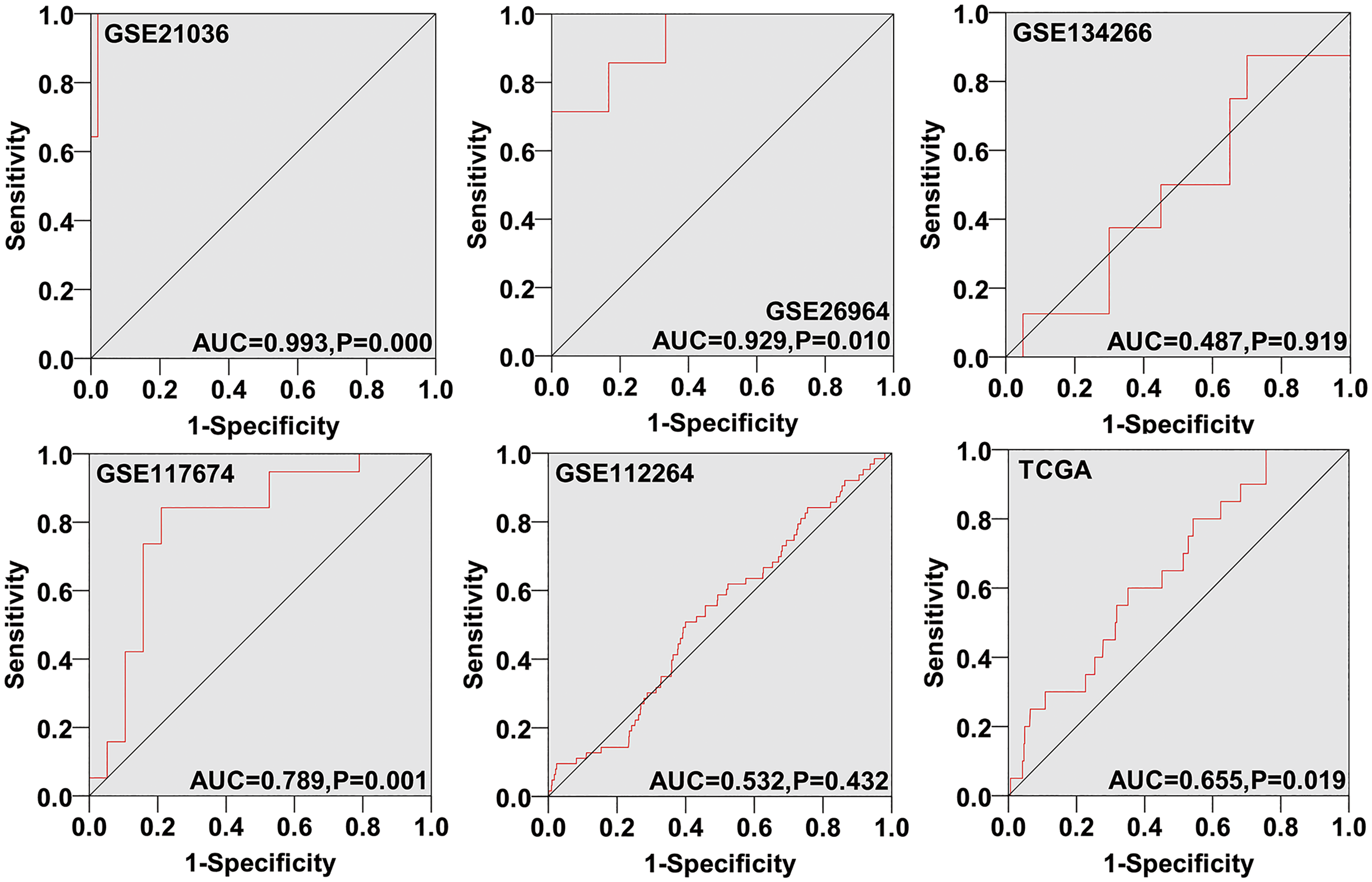

Supplement: Supplementary file 3 — Supplementary material 3 [file SYB2-15-1-s003.tif]

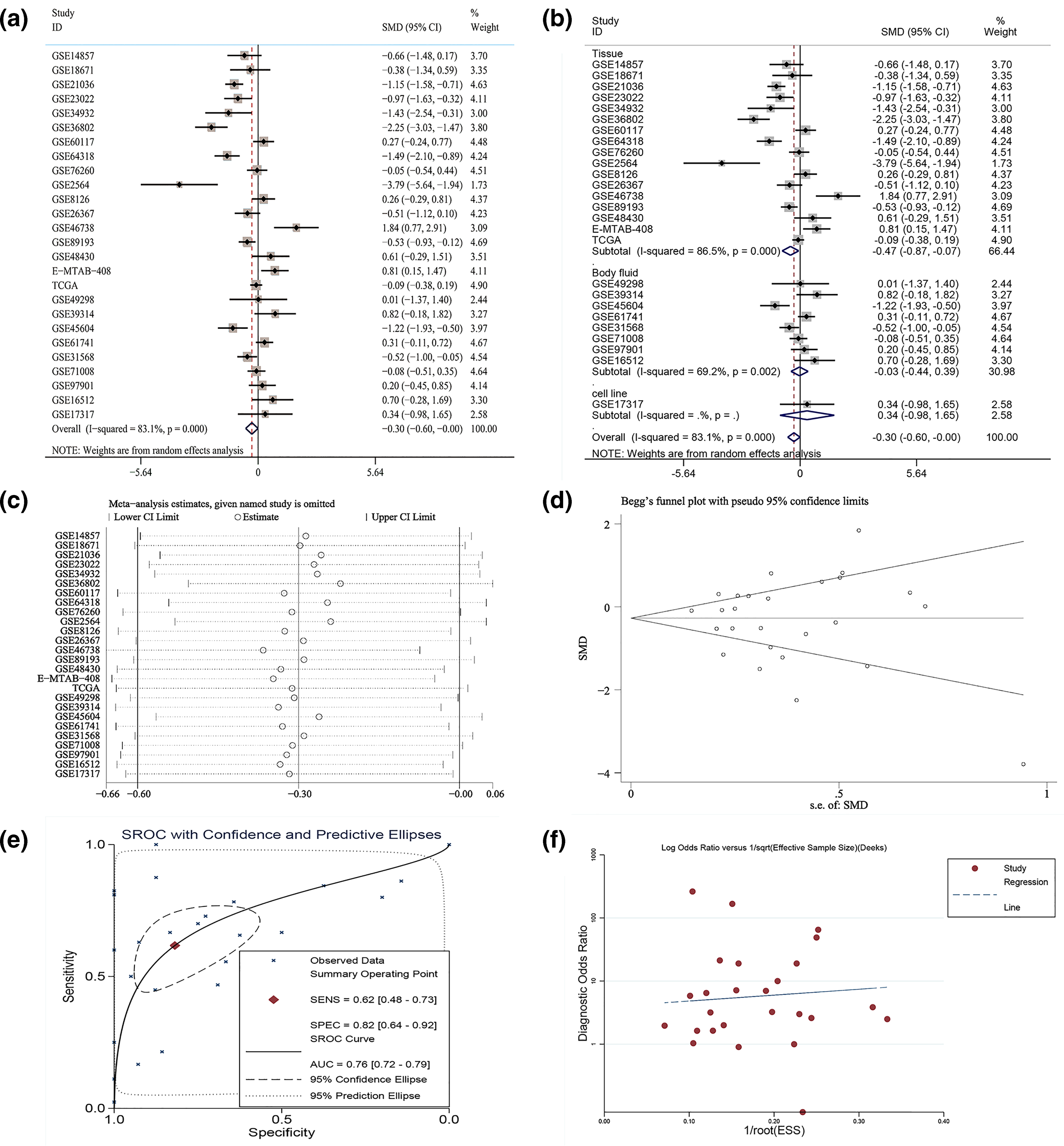

Supplement: Supplementary file 4 — Supplementary material 4 [file SYB2-15-1-s004.tif]

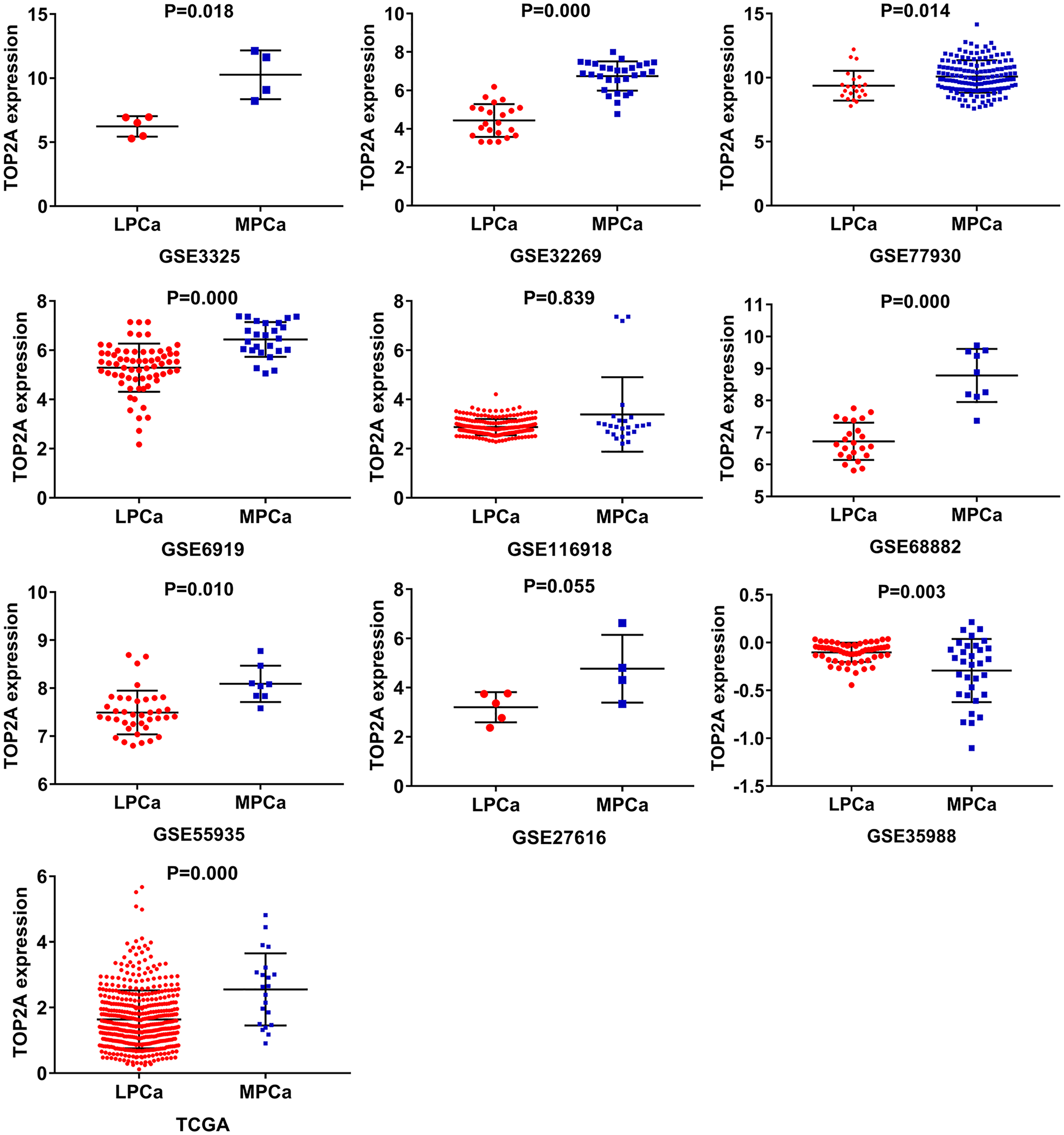

Supplement: Supplementary file 5 — Supplementary material 5 [file SYB2-15-1-s005.tif]

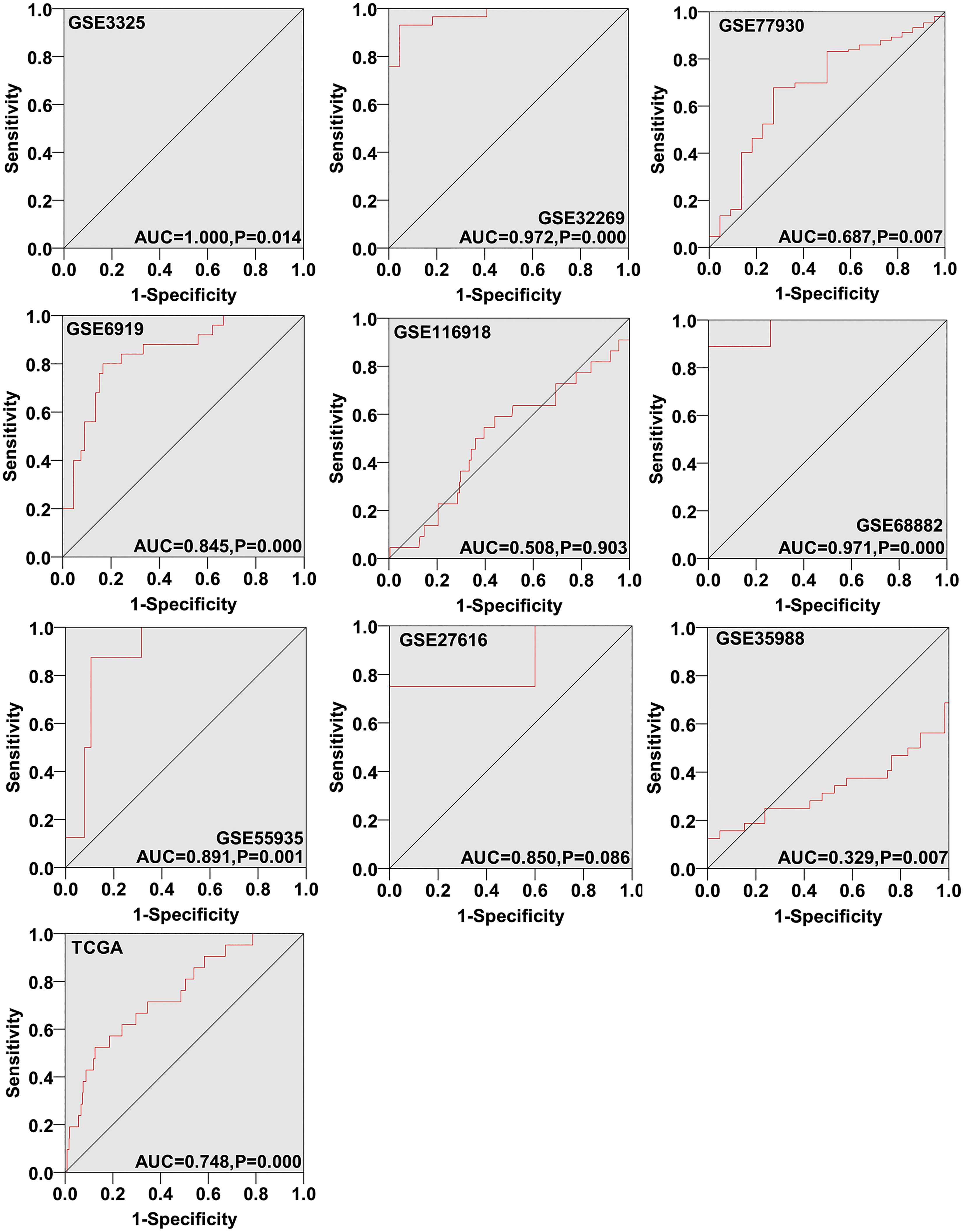

Supplement: Supplementary file 6 — Supplementary material 6 [file SYB2-15-1-s006.tif]

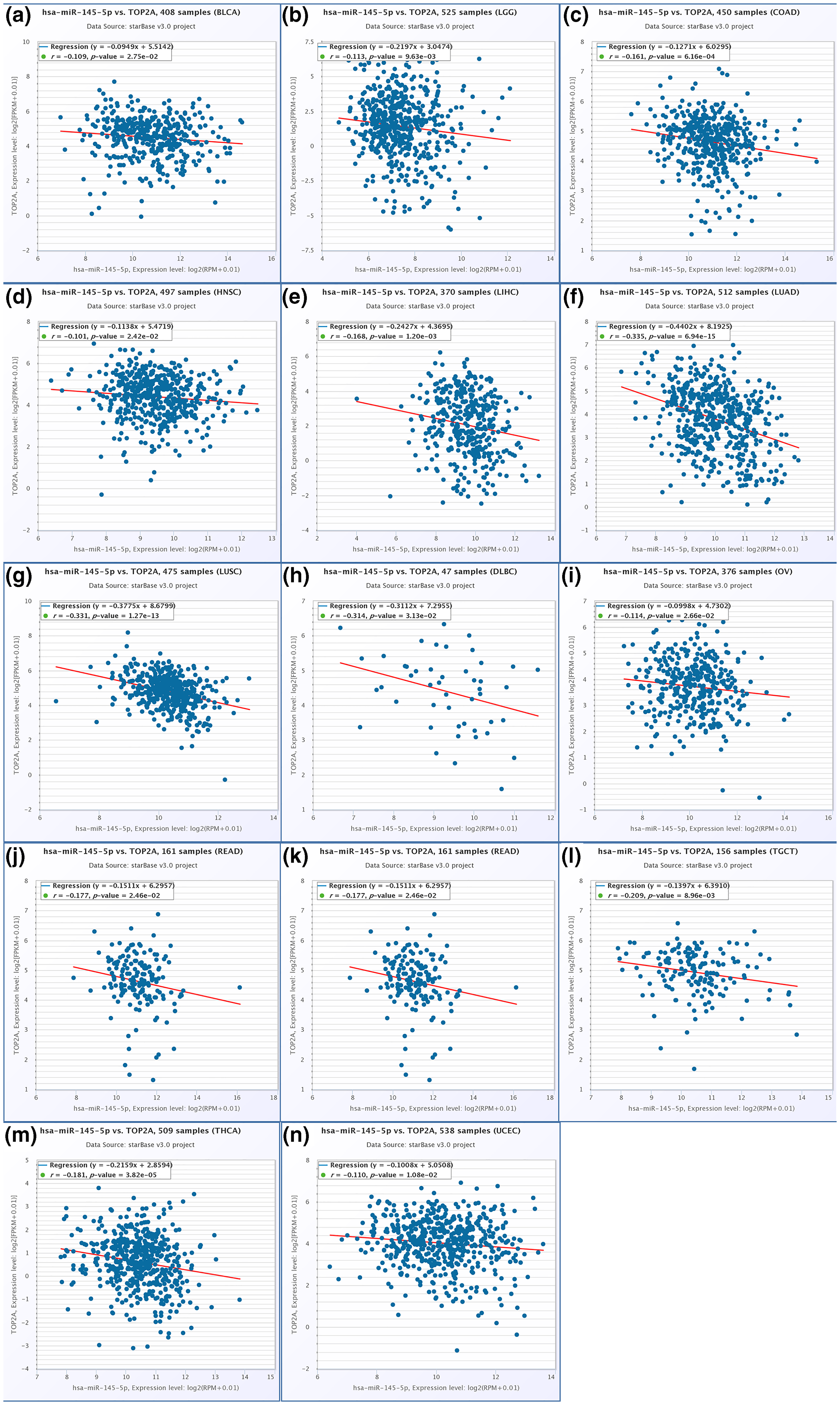

Supplement: Supplementary file 7 — Supplementary material 7 [file SYB2-15-1-s007.tif]

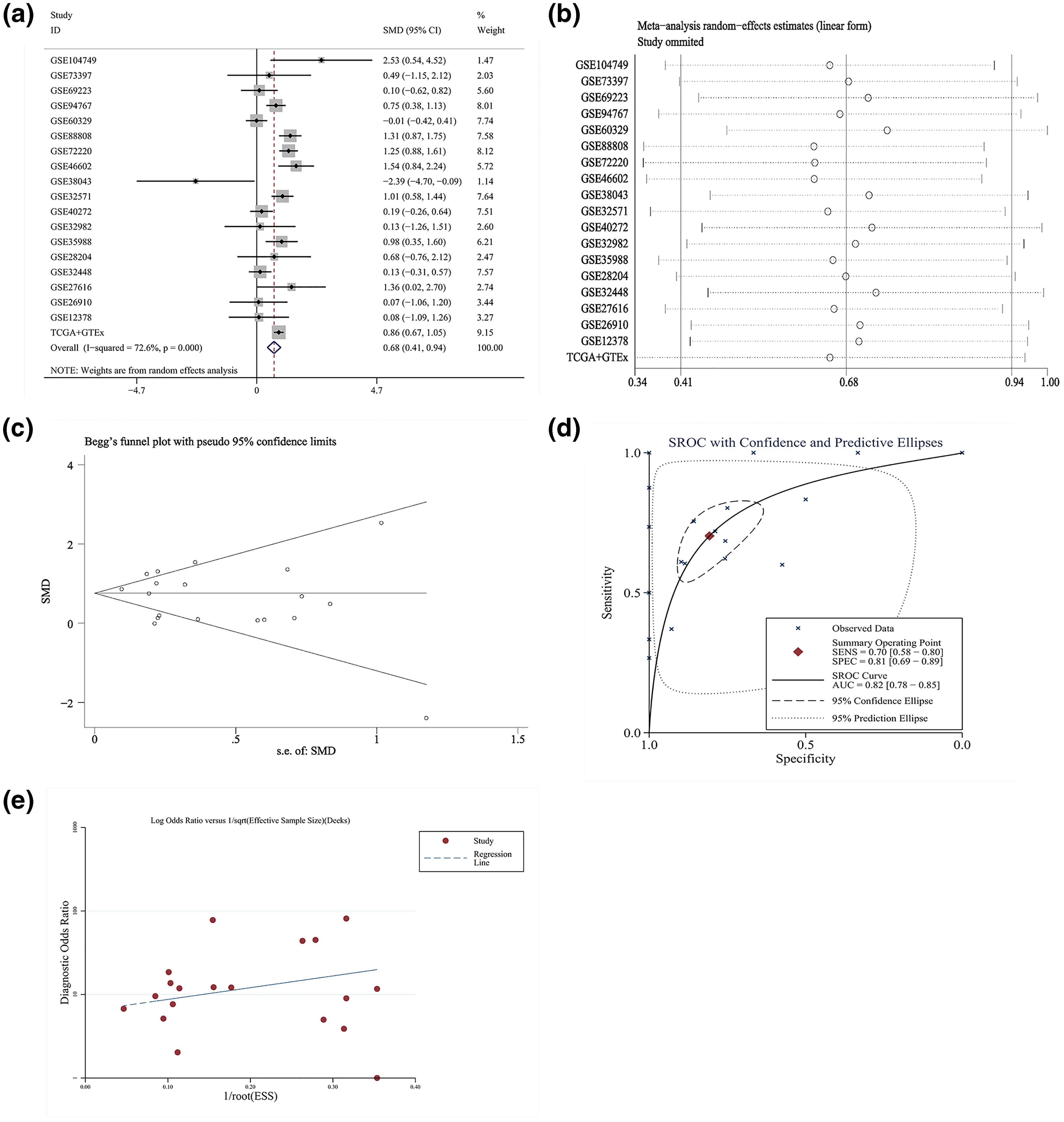

Supplement: Supplementary file 8 — Supplementary material 8 [file SYB2-15-1-s008.tif]
