# Supplementary material for: MiRNA‐145‐5p expression and prospective molecular mechanisms in the metastasis of prostate cancer
Source: IET Syst Biol. 2021 Feb 1;15(1):1–13. doi: 10.1049/syb2.12011 (PMC8675798; doi:10.1049/syb2.12011)
Supplement: Supplementary file 9 — Supplementary material 9 [file SYB2-15-1-s011.docx]

Supplemental Table S1. The means and standard deviations of miR-145-5p expression values in PCa and non-PCa based on 26 studies.

| Study | Country | Year | Sample | PCa | | |  | Non-PCa | | |
| --- | --- | --- | --- | --- | --- | --- | --- | --- | --- | --- |
|  |  |  | type | N | M | SD |  | N | M | SD |
| GSE14857 | Germany | 2009 | Tissue | 12 | 15.636 | 1.116 |  | 12 | 16.213 | 0.543 |
| GSE18671 | Germany | 2011 | Tissue | 14 | 9.956 | 1.226 |  | 6 | 10.669 | 2.978 |
| GSE21036 | USA | 2010 | Tissue | 114 | 13.690 | 1.544 |  | 28 | 15.300 | 0.465 |
| GSE23022 | Germany | 2010 | Tissue | 20 | 4.675 | 0.274 |  | 20 | 4.918 | 0.222 |
| GSE34932 | China | 2012 | Tissue | 8 | 8.890 | 2.012 |  | 8 | 11.061 | 0.764 |
| GSE36802 | USA | 2013 | Tissue | 21 | 9.240 | 0.935 |  | 21 | 10.865 | 0.414 |
| GSE60117 | Italy | 2014 | Tissue | 56 | 12.411 | 0.739 |  | 21 | 12.225 | 0.572 |
| GSE64318 | USA | 2014 | Tissue | 27 | 11.520 | 0.775 |  | 27 | 12.532 | 0.563 |
| GSE76260 | Italy | 2015 | Tissue | 32 | 14.385 | 0.695 |  | 32 | 14.416 | 0.596 |
| GSE2564 | USA | 2012 | Tissue | 6 | 5.267 | 0.457 |  | 8 | 7.589 | 0.703 |
| GSE8126 | USA | 2012 | Tissue | 60 | 14.113 | 0.755 |  | 16 | 13.902 | 0.986 |
| GSE26367 | USA | 2012 | Tissue | 173 | 14.388 | 0.493 |  | 11 | 14.647 | 0.686 |
| GSE46738 | Brazil | 2013 | Tissue | 53 | 7.861 | 0.349 |  | 4 | 7.230 | 0.233 |
| GSE89193 | USA | 2016 | Tissue | 49 | 12.558 | 1.498 |  | 49 | 13.296 | 1.296 |
| GSE48430 | USA | 2014 | Tissue | 10 | 20.759 | 2.525 |  | 10 | 19.310 | 2.237 |
| E-MTAB-408 | Finland | 2012 | Tissue | 42 | 13.267 | 0.959 |  | 12 | 12.508 | 0.870 |
| TCGA | NA | NA | Tissue | 498 | 12.184 | 0.821 |  | 52 | 12.258 | 0.683 |
| GSE49298 | Turkey | 2013 | Body fluid | 4 | 4.226 | 0.341 |  | 4 | 4.221 | 0.382 |
| GSE39314 | Mexico | 2012 | Body fluid | 9 | 8.349 | 2.589 |  | 8 | 6.549 | 1.632 |
| GSE45604 | Spain | 2014 | Body fluid | 50 | 19.648 | 2.861 |  | 10 | 23.270 | 3.524 |
| GSE61741 | Germany | 2014 | Body fluid | 65 | 7.372 | 1.523 |  | 35 | 6.864 | 1.870 |
| GSE31568 | Germany | 2011 | Body fluid | 23 | 4.361 | 2.196 |  | 70 | 5.411 | 1.948 |
| GSE71008 | USA | 2015 | Body fluid | 36 | 6.408 | 0.221 |  | 50 | 6.426 | 0.227 |
| GSE97901 | USA | 2017 | Body fluid | 32 | 5.677 | 0.752 |  | 13 | 5.507 | 1.049 |
| GSE16512 | USA | 2009 | Body fluid | 6 | 7.342 | 1.465 |  | 14 | 6.637 | 0.749 |
| GSE17317 | Germany | 2009 | cell line | 9 | 9.665 | 0.203 |  | 3 | 9.600 | 0.123 |
| miR, microRNA; PCa, prostate cancer; N, number; M, mean; SD, standard deviation; TCGA, The Cancer Genome Atlas. | | | | | | | | | | |
