# Supplementary material for: MiRNA‐145‐5p expression and prospective molecular mechanisms in the metastasis of prostate cancer
Source: IET Syst Biol. 2021 Feb 1;15(1):1–13. doi: 10.1049/syb2.12011 (PMC8675798; doi:10.1049/syb2.12011)
Supplement: Supplementary file 10 — Supplementary material 10 [file SYB2-15-1-s012.docx]

Supplemental Table S2. Association between miRNA-145-5p expression and clinicopathological parameters in PCa samples based on TCGA database.

| Clinicopathological |  | miRNA-145-5p expression | | | T-test |  |
| --- | --- | --- | --- | --- | --- | --- |
| parameters | N | M | SD |  | T-value | P-value |
| Group |  |  |  |  |  |  |
| Non-cancer | 52 | 12.258 | 0.683 |  | 0.628 | 0.530 |
| Cancer | 498 | 12.184 | 0.821 |  |  |  |
| Age (years) |  |  |  |  |  |  |
| < 60 | 195 | 12.241 | 0.830 |  | 1.430 | 0.153 |
| ≥ 60 | 287 | 12.130 | 0.842 |  |  |  |
| Pathological T stage |  |  |  |  |  |  |
| T1+T2 | 194 | 12.377 | 0.849 |  | 4.350 | 0.000 |
| T3+T4 | 301 | 12.047 | 0.797 |  |  |  |
| N stage |  |  |  |  |  |  |
| N0 | 348 | 12.184 | 0.807 |  | 2.890 | 0.004 |
| N1 | 78 | 11.894 | 0.770 |  |  |  |
| M stage |  |  |  |  |  |  |
| M0 | 481 | 12.914 | 0.827 |  | 2.510 | 0.012 |
| M1 | 20 | 11.721 | 0.827 |  |  |  |
| Gleason score |  |  |  |  |  |  |
| ≤ 7 | 291 | 12.311 | 0.815 |  | 4.441 | 0.000 |
| 8 ≥ | 203 | 11.979 | 0.821 |  |  |  |
| Recurrence |  |  |  |  |  |  |
| No | 437 | 12.207 | 0.838 |  | 2.253 | 0.025 |
| Yes | 58 | 11.946 | 0.763 |  |  |  |

M: mean; N: number; PCa: prostate cancer; SD: standard deviation; TCGA: The Cancer Genome Atlas.
