# Supplementary material for: MiRNA‐145‐5p expression and prospective molecular mechanisms in the metastasis of prostate cancer
Source: IET Syst Biol. 2021 Feb 1;15(1):1–13. doi: 10.1049/syb2.12011 (PMC8675798; doi:10.1049/syb2.12011)
Supplement: Supplementary file 11 — Supplementary material 11 [file SYB2-15-1-s010.docx]

Supplemental Table S3. The means and standard deviations of TOP2A expression values for PCa and non-PCa based on 19 studies.

| Study | Country | Year | Sample | PCa | | |  | Non-PCa | | |
| --- | --- | --- | --- | --- | --- | --- | --- | --- | --- | --- |
|  |  |  | type | N | M | SD |  | N | M | SD |
| GSE104749 | China | 2017 | tissue | 4 | 7.421 | 0.743 |  | 4 | 3.892 | 1.827 |
| GSE73397 | China | 2015 | tissue | 3 | 9.109 | 0.644 |  | 3 | 8.675 | 1.084 |
| GSE69223 | Germany | 2015 | tissue | 15 | -0.241 | 1.713 |  | 15 | -0.383 | 1.060 |
| GSE94767 | United Kingdom | 2017 | tissue | 185 | 5.640 | 0.939 |  | 33 | 4.965 | 0.604 |
| GSE60329 | Italy | 2014 | tissue | 108 | -0.038 | 1.057 |  | 28 | -0.029 | 0.665 |
| GSE88808 | USA | 2016 | tissue | 49 | 7.389 | 0.900 |  | 49 | 6.365 | 0.642 |
| GSE72220 | USA | 2015 | tissue | 57 | -0.007 | 0.130 |  | 90 | -0.127 | 0.068 |
| GSE46602 | Denmark | 2013 | tissue | 34 | 4.181 | 0.979 |  | 14 | 2.867 | 0.377 |
| GSE38043 | USA | 2012 | body fluid | 3 | 6.033 | 0.361 |  | 3 | 7.144 | 0.548 |
| GSE32571 | Germany | 2011 | tissue | 59 | 7.020 | 0.616 |  | 39 | 6.500 | 0.310 |
| GSE40272 | USA | 2012 | tissue | 43 | -0.123 | 0.588 |  | 35 | -0.254 | 0.801 |
| GSE32982 | Finland | 2011 | tissue | 6 | 4.586 | 0.490 |  | 3 | 4.532 | 0.136 |
| GSE35988 | USA | 2012 | tissue | 76 | 3.038 | 1.557 |  | 12 | 1.583 | 0.932 |
| GSE28204 | China | 2011 | tissue | 4 | 7.498 | 3.012 |  | 4 | 5.733 | 2.092 |
| GSE32448 | USA | 2011 | tissue | 40 | 3.162 | 0.909 |  | 40 | 3.039 | 0.958 |
| GSE27616 | USA | 2011 | tissue | 8 | 3.298 | 1.257 |  | 4 | 1.719 | 0.901 |
| GSE26910 | Italy | 2011 | tissue | 6 | 3.042 | 0.408 |  | 6 | 3.018 | 0.282 |
| GSE12378 | United Kingdom | 2008 | tissue | 36 | 5.238 | 0.844 |  | 3 | 5.167 | 1.129 |
| TCGA+GTEx | NA | NA | tissue | 492 | 1.678 | 0.908 |  | 152 | 0.931 | 0.718 |

PCa, prostate cancer; N, number; M, mean; SD, standard deviation; TCGA, The Cancer Genome Atlas; GTEx, The Genotype-Tissue Expression.
