# Supplementary material for: MiRNA‐145‐5p expression and prospective molecular mechanisms in the metastasis of prostate cancer
Source: IET Syst Biol. 2021 Feb 1;15(1):1–13. doi: 10.1049/syb2.12011 (PMC8675798; doi:10.1049/syb2.12011)
Supplement: Supplementary file 12 — Supplementary material 12 [file SYB2-15-1-s009.docx]

Supplemental Table S4. Association between TOP2A expression and clinicopathological parameters in PCa samples based on TCGA database.

| Clinicopathological |  | TOP2A expression | |  | T-test |  |
| --- | --- | --- | --- | --- | --- | --- |
| parameters | N | M | SD |  | T-value | P-value |
| Group |  |  |  |  |  |  |
| Non-cancer | 52 | 0.8181 | 72436 |  | -9.519 | 0 |
| Cancer | 498 | 1.6782 | 0.90836 |  |  |  |
| Age (years) |  |  |  |  |  |  |
| < 60 | 195 | 1.5721 | 0.83007 |  | -2.125 | 0.034 |
| ≥ 60 | 287 | 1.7517 | 0.96198 |  |  |  |
| Pathological T stage |  |  |  |  |  |  |
| T1+T2 | 194 | 1.3102 | 0.6577 |  | -7.43 | 0 |
| T3+T4 | 301 | 1.9013 | 0.95623 |  |  |  |
| N stage |  |  |  |  |  |  |
| N0 | 348 | 1.6395 | 0.84797 |  |  |  |
| N1 | 78 | 2.1703 | 1.01516 |  | -4.804 | 0 |
| M stage |  |  |  |  |  |  |
| M0 | 478 | 1.6397 | 0.88055 |  | -4.601 | 0 |
| M1 | 21 | 2.5533 | 1.10161 |  |  |  |
| Gleason score |  |  |  |  |  |  |
| ≤ 7 | 291 | 1.3724 | 0.68223 |  | -9.696 | 0 |
| 8 ≥ | 203 | 2.1077 | 1.00506 |  |  |  |
| Recurrence |  |  |  |  |  |  |
| No | 437 | 1.6129 | 0.87643 |  | -4.293 | 0 |
| Yes | 58 | 2.1467 | 0.98573 |  |  |  |

M: mean; N: number; PCa: prostate cancer; SD: standard deviation; TCGA: The Cancer Genome Atlas.
